# Up-regulation of CatSper genes family by selenium

**DOI:** 10.1186/1477-7827-7-126

**Published:** 2009-11-16

**Authors:** Shabnam Mohammadi, Mansoureh Movahedin, Seyed Javad Mowla

**Affiliations:** 1 Department of Anatomy, Faculty of Medical Sciences, Tarbiat Modares University, Tehran, Iran; 2 Department of Genetics, Faculty of Basic Sciences, Tarbiat Modares University, Tehran, Iran

## Abstract

**Background:**

CatSper1-4 are a unique family of sperm cation channels, which are exclusively expressed in the testis and play an important role in sperm motility and male fertility. Despite their vital role in male fertility, almost nothing is known about the factors regulating their expression. Here, we investigated the effects of selenium (Se) on the expression of CatSper genes and sperm parameters in aging versus young male mice.

**Methods:**

Forty 11-13 months aging male mice and forty 2-3 months young adult male mice were used. The animals were divided in two experimental groups: first group including aging males and second group comprising of young adult males, both treated with Se. The experimental groups were injected intra-peritoneally with Se (0.2 mg/kg) daily, for up to 5 weeks. Two other groups, aging and young adult mice without Se treatment were used as controls. All the animals were rapidly sacrificed by cervical dislocation on the days 21, 28, 35 and 42 after Se treatment. Subsequently, the morphology of the collected sperms was analyzed, and one of the testes from each mouse used for semi-quantitative RT-PCR. The significancy of the data was analyzed using ANOVA.

**Results and Discussion:**

Our data revealed that there was a significant up-regulation of CatSper genes in the experimental groups compared to the control ones. Furthermore, the results of sperm analysis showed that the sperm parameters were improved in the aging as well as young adult male mice following Se treatment.

**Conclusion:**

Se treatment in the aging subjects could up-regulate the expression of CatSper genes, and therefore results in elevation of sperm motility. Furthermore, Se treatment improved sperm parameters, especially morphology and viability rates.

## Background

Calcium channels play important roles in the functions of sperm such as motility, capacitation, and acrosome reaction [1]. Many calcium-permeable channels have been identified in mammalian sperm, including voltage-gated calcium channels, cyclic nucleotide-gated channels, and transient receptor potential channels [1]. *CatSper *genes (1-4) are specific sperm calcium channels, which are expressed merely in the testis and are required for sperm motility and male fertility [2]. *CatSper *genes expression in mouse is detected as early as 3 weeks of age [3,4]. *CatSper1 *was cloned and characterized by Ren et al. in 2001 [5]. The protein is localized to the principal piece of sperm tail, and plays an important role in sperm motility, penetration into oocyte, and male fertility [5]. *CatSper2 *is critical to sperm hyperactivation motility and male fertility [6]. Lobely et al. have reported the other two members of the *CatSper *family, *CatSper3 *and *CatSper4 *[7]. The latter genes are expressed within the acrosome of late spermatids and spermatozoa and play an important role in acrosome reaction and male fertility [8].

In aging mice, histological changes of the testis, the decrease in sperm motility and gene expression suggest that oxidative stress is correlated with the aging process [9]. The production of extensive rates of reactive oxygen species (ROS) in semen results in oxidative stress [10]. The seminal plasma contains a variety of antioxidants, which includes glutathione peroxidase, catalase, superoxide dismutase, vitamin C, vitamin E, pyruvate, and carnitine that protect sperms against ROS [10].

Selenium (Se) plays a crucial role in the maintenance of reproduction, partly because it is an essential component of glutathione peroxidase [11]. In addition, testis is the main target organ for Se, and its level remarkably increases during puberty [11].

Wu et al. have reported that the Se-deficiency had caused seminiferous tubule degeneration as well as a reduced number of spermatozoa with a lower motility [12]. Furthermore, flagellar bends were detected in Se-deficient mice [13]. In some infertile men, ultrastructural defects were reported in midpiece's mitochondria [13]. Moreover, excess amounts of Se supplements seem to have a negative effect. For example, rats fed with 4 ppm Se for 5 weeks produced spermatozoa with impaired motility and morphological abnormalities, especially in the midpiece of sperm [11]. In rats with Se-enriched diet, a reduction in tubular diameters, epithelial height, number of spermatogenic cells as well as an increase in the amount of abnormal spermatozoa have been reported [11]. Based on the latter report, the effect of Se on sperm parameters seems to be time- and dose-dependent [11].

Direct effect of sperm motility on fertility as well as decrease in sperm parameters with increase of age have been already reported in many studies [14]. Since *CatSper *is one of the responsible genes for producing sperm motility, we attempted to answer the following questions: Firstly, do *CatSper *genes expressions decrease with aging in mice testis? Secondly, does Se administration increases *CatSper *genes expression in mice testis?

In the present study, the effect of Se on *CatSper *genes expression was investigated for the first time. We focused on *CatSper *genes expression in 11-13 months aging and 2-3 months young adult male mice before and after Se treatment. We also analyzed sperm parameters after Se treatment in the aging and young adult male mice.

## Methods

### Chemical

Sodium selenite (Na_2_Seo_3_) was purchased from Sigma-Aldrich Co (USA).

### Animals

Forty 11-13 months old aging male NMRI mice and forty 2-3 months old young adult male mice were purchased from the Pasteur Institute (Tehran, Iran). The mice were housed in a room under standard laboratory conditions (12 h light, 12 h dark at 22°C) with free access to water and food. The mice were randomly divided into four groups (control 1: aging male without Se treatment, control 2: young adult male without Se treatment, Experimental 1: aging male treated with Se and Experimental 2: young adult male treated with Se). The experimental groups were injected intra-peritoneally with Se at 0.2 mg/kg (based on dose/response data); daily for 42 days. The animals were rapidly sacrificed by cervical dislocation on the days 21, 28, 35, 42 after the treatment. One testis and epididymis were collected from each group. The testis from each mouse was separately and immediately frozen in liquid nitrogen and stored at -80°C for subsequent RNA extraction.

### RNA extraction and reverse transcription

Total RNA from the testis was extracted using RNX plus solution (Cinnagen, Iran) according to the manufacturer's instructions. The tissue was grinded to a powder on dry ice, lysed with 1 ml of RNX solution and incubated at room temperature for 5 min. Chloroform was added to the solution and centrifuged at 12000 g, at 4°C, for 15 min. The upper phase was transferred into a tube and an equal volume of isopropanol was added to the solution. The tube was then placed at 4°C for 30 min and centrifuged at 12000 g, for 15 min at 4°C. The pellet was washed with 75% ethanol and dissolved in RNase-free water. The purity and integrity of RNA were checked by 260/280 nm ratio measurement and 1% agarose gel electrophoresis, respectively. The total RNA (1 μg) was reverse transcribed with 200 U/μl MMLV reverse transcriptase, 20 mM dNTP, 20 U/μl RNase inhibitor (RNasin) and oligo (dt)_18 _priming in a 20 μl reaction. The samples were incubated at 42°C for 60 min, then the transcriptase was inactivated at 70°C for 10 min. All reverse transcription (RT) reagents were purchased from Fermentas Corporation (Germany).

### Polymerase Chain Reaction (PCR)

The primers specific to *CatSper1-4 *and β2m genes were designed using previously described mouse described sequences (GenBank) and Genrunner software (version 3.02; Hastings Software), as shown in Table 1. β2-microglobulin (β2-m), a housekeeping gene, was included as an internal control to normalize the PCR reaction. The PCR was done using the BIOTECH-MWG Thermocycler (Germany) in a total volume of 50 μl. PCR master mix contained: 2 μl of synthesized CDNA, 3 μl (25 Mm) MgCl_2_, 0.25 Taq DNA polymerase, 5 μl 10× PCR buffer, 1 μl 20 mM dNTP_S_, 1.5 μl (20 pmol) forward primer, 1.5 μl reverse primer, and 35.75 μl of water. The PCR conditions were: 94°C for 2 min followed by 35 cycles (94°C for 30 s, 58°C for 30 s and 72°C for 1 min), and a final extension at 72°C for 10 min.

**Table 1:** The sequence of the designed primers used for RT-PCR

Gene	Genebank	Reverse primer	Forward primer	Product size
*CatSper 1*	AF407332	5'CACACACCGGGAATATCTTC3'	5'TCGGAGAACCACAGAGAAGAG3'	566 bp
*CatSper 2*	AF411816	5'TGTCAGGCTGTTGCTTTGTC3'	5'TGGCCACAGAGCAGTATTTG3'	513 bp
*CatSper 3*	AK014942	5'GCTCTTCCTCCTCATGTTTG3'	5'TCTTCCAACATCAGGCTCAG3'	597 bp
*CatSper 4*	AK077145	5'AAGGGGACACAGCAAAGATG3'	5'TATTCCAGCCATCCTTCCAG3'	417 bp
β2m	NM-009735	5'TGACCGGCTTGTATGCTATC3'	5'CACATGTCTCGATCCCAGTAG3'	316 bp

### Semi-quantitative RT-PCR

The PCR products were analyzed on %1.5 agarose gel (Gibco BRL) and visualized under ultraviolet transillumination after staining with ethidium bromide. RT-PCR reaction was performed for both *CatSper *and β2m genes and the intensity of each band was quantified using Uvidoc software (version 12.4, England). The ratio of the *CatSper *genes bands intensity were compared to the corresponding β2m. All PCRs were independently replicated three times.

### Sperm analysis

Sperm parameters (count, motility, morphology and viability rates) were analyzed according to the WHO guidelines given for human sperm examination. Epididymis was finely minced in PBS and incubated at 37°C for 30-45 minutes. The count and motility of the sperm were evaluated using a Neubaur haemocytometer under a 400× magnification light microscopy and the viability rate was assessed using vital staining.

### Statistical analysis

The results were expressed as the mean ± SD. The data were analyzed using ANOVA and the results were assumed significant when p < 0.05.

## Results

### Effects of selenium on *CatSper *genes expression in the aging male mice

**Figure 1:**
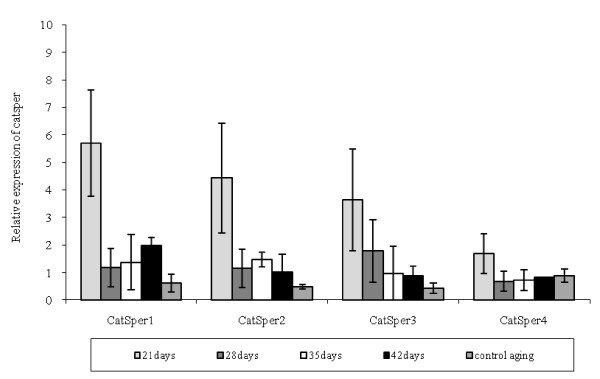
**Comparison between the relative gene expression of *CatSper*s and β2m in the aging mice testes**. The values are shown as the mean ± SD. The experiments were replicated at least3 times.

**Figure 2:**
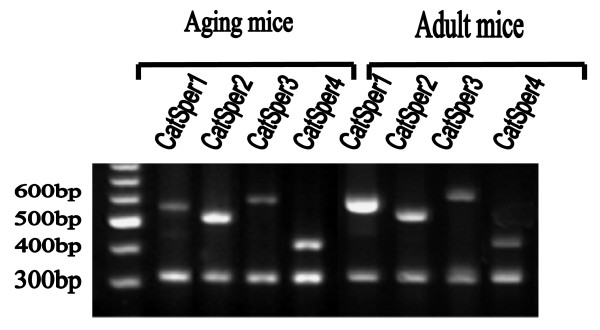
**RT-PCR analysis of the *CatSper *genes expression in the aging and young adult mice testes 35 days after selenium treatment**. The experiments were at least replicated 3 times.

As shown in Figure 1, the relative intensity of *CatSper1 *expression in the experimental group 1 was significantly higher than that in the control 1, on the day 21 of Se treatment (5.70 ± 1.1 vs. 0.61 ± 0.32, respectively). The expression was then decreased on the days 28 and 35 (Figure 2), and again began to increase on the day 42. The same results were obtained for *CatSper*3 expression. The relative intensity of *CatSper2 *expression showed his peak on the day 21, declined to its lowest level on the day 28, then increased on the day 35 and once more decreased on the day 42. The relative intensity of *CatSper4 *expression was not significant in the control group on the day 21 (1.68 ± 0.73 vs. 0.89 ± 0.24 for the experimental 1 and control 1 groups, respectively). *CatSper4 *expression declined until the day 28 and then increased on the days 35 and 42.

### Effects of Se on *CatSper *genes expression in the adult male mice

**Figure 3:**
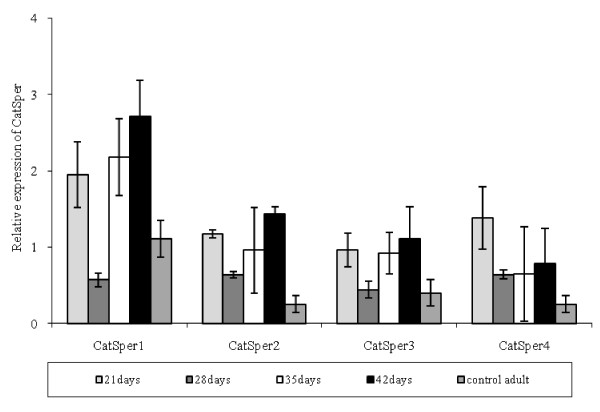
**Comparison between the relative gene expression of *CatSper*s and β2m in the young adult mice testes**. values are shown as the mean ± SD. The experiments were replicated at least 3 times.

As shown in Figure 3, the relative intensity of *CatSper1 *expression in the experimental group 2 significantly increased, compared to that in the control group 2 (1.95 ± 0.43 vs. 1.11 ± 0.24, respectively) and then declined on the day 28. Its expression was significantly more than that of the control group 2 on the day 35 (2.18 ± 0.26 vs. 1.11 ± 0.24 for experimental 2 and control 2 groups, respectively); the expression again decreased until the day 42. The relative intensity of *CatSper2 *expression in the experimental group 2 was significantly more than that in the control 1 on the day 21 (1.17 ± 0.05 vs. 0.25 ± 0.11 for the experimental 2 and the control 2 groups, respectively), which was decreased on the day 28 and began to increase again on the days 35 (Figure 1) and 42. Similar results were found for *CatSper3 *expression. The relative intensity of *CatSper4 *expression peaked on the day 21, then dropped to its lowest level on the days 28, 35 and again increased on the day 42.

## Discussion

In our previous study, we have shown that sperm parameters in aging mice decreased, compared to those of the young adult mice [15]. Many mechanisms have been suggested to explain the effects of aging on sperm quality [14]. For example, in the aging subjects, smooth muscle atrophy in the prostate and a decrease in its protein and water content may affect semen volume and sperm motility. Sperm acquires capacitation when it passes through the epididymis, where it plays an important role in sperm maturation. Sperm motility decreases during aging because of suppression in epididymal function. Sperm morphology is a good index for the status of the germinal epithelium. Aging causes degenerative alterations in the germinal epithelium and may affect spermatogenesis and alter sperm morphology [14].

*CatSper *genes expression in mouse testis was detected as early as 3 weeks of age by Nikpoor et al [4]. In the present study, *CatSper *gene family was also expressed in the 2-3 months old young adult and the 11-13 months old aging male mice and no significant decrease was detected in the expression of *CatSper *genes between the two groups. A novel finding of our study is that Se administration caused up-regulation in *CatSper *genes family. However, Se treatment in the 11-13 months old aging male mice caused more up-regulation compared to the young adult ones, suggesting that the Se treatment had a more profound effect on *CatSper *expression in the aging male mice. At the same time, spermatogenesis was improved in the aging subjects treated with Se. It seems that there is a correlation between Se treatment and improvement of sperm quality. Noting that *CatSper1 *gene expression changes were more than those of the other *CatSper *family members, most likely this gene has a more strong regulatory effect (at least for Se). However, the expression of *CatSper1 *was not changed with an increase in age. Furthermore, we observed a significant up-regulation of *CatSper1 *gene expression on the day 21 in the aging male mice, showing that short-time Se treatment was effective. The relative intensity of *CatSper *genes expression in the aging mice was more than that in the young adult mice on the days 21 and 28, and the ratio in the adult mice was more than that in the aging mice on the days 35 and 42. However, further molecular studies are needed to confirm our finding. Regulation of *CatSper *genes factors and the effects of antioxidants on their expression have not been reported thus far. Some studies have demonstrated that antioxidants could affect gene expression. Gan et al [16]. observed that GSH-PX mRNA levels remarkably increased in the rats testis injected with 0.02 mg/kg/d Se. However, injection of 0.04 and 0.08 mg/kg Se dramatically decreased GSH-PX expression, in comparison to the 0.02 mg/kg group. In another study, Jervis et al. reported that vitamin E treatment affected the expression of oxidative stress-related genes in the aging male rats. Moreover, vitamin E deficiency causes the accumulation of oxidative stress damage in the epididymis of aging rats [9]. A recent study has demonstrated that changes in the Se level leads to a decline in the expression pattern of both c-Jun and c-Fos genes, which might be responsible for the decline in the number and differentiation of spermatogonia, resulting in the reduction of fertility [17].

## Conclusion

Se treatment in the aging subjects could up-regulate the expression of *CatSper *genes, which are among the genes responsible for sperm motility. Besides, Se treatment improved sperm parameters especially its morphology and viability rates.

## Competing interests

The authors declare that they have no competing interests.

## Authors' contributions

SM carried out the molecular genetic studies and participated in design of the study and in drafted the manuscript. MM participated in the design of the study and participated in drafted the manuscript. SJM participated in its design and helped to draft the manuscript. All authors read and approved the final manuscript.

## References

[B1] Darszon A Lopez-Martinez P Acevedo JJ Hernandez-Cruz A Trevino CL T-type Ca^2+ ^channels in sperm function Cell Calcium 2006 40 241 252 10.1016/j.ceca.2006.04.028 16797697

[B2] Qi H Moran MM Navarro B Chong JA Krapivinsky G Krapivinsky L Kirichok Y Ramsey IS Quill TA Clapham DE All four CatSper ion channel proteins are required for male fertility and sperm cell hyperactivated motility PNAS 2007 104 4 1219 1223 17227845 10.1073/pnas.0610286104 PMC1770895

[B3] Asadi MH Mowla SJ Nikpoor P Gene expression profile of CatSper3 and CatSper4 during postnatal development of mouse testis IBJ 2006 10 111 115

[B4] Nikpoor P Mowla SJ Movahedin M Ziaee SA Taki T CatSper gene expression in postnatal development of mouse testis and in subfertile men with deficient sperm motility Human Reprod 2004 19 124 128 10.1093/humrep/deh043 14688170

[B5] Ren D Navarro B Perez G Jackson AC Hsu S Shi Q Tilly JL Clapham DE A sperm ion channel required for sperm motility and male fertility Nature 2001 413 603 609 10.1038/35098027 11595941 PMC8462998

[B6] Quill TA Sugden SA Rossi KL Doolittle LK Hammer RE Garbers DL Hyperactivated sperm motility driven by CatSper2 is required for fertilization PNAS 2003 100 14869 14874 14657366 10.1073/pnas.2136654100 PMC299835

[B7] Lobley A Pierron V Reynolds L Allen L Michalovich D Identification of human and mouse CatSper3 and CatSper4 genes: Characterisation of a common interaction domain and evidence for experssion in testis Reprod Biol Endocrinol 2003 1 53 67 12932298 10.1186/1477-7827-1-53 PMC184451

[B8] Jin JL Odoherty AM Wang S Zheng H Sandres KM Yan W CatSper3 and CatSper4 encode two cation channel-like proteins exclusively expressed in the testis Biol Reprod 2005 73 1235 1242 10.1095/biolreprod.105.045468 16107607

[B9] Jervis KM Robaire B The effects of long-term vitamin E treatment on gene expression and oxidative stress damage in the aging brown Norway rat epididymis Biol Reprod 2004 71 1088 1095 10.1095/biolreprod.104.028886 15175234

[B10] Agarwal A Nallella KP Allamaneni SR Said TM Role of antioxidant in treatment of male infertility: an overview of the literature RBM Online 2004 8 616 627 15169573 10.1016/s1472-6483(10)61641-0

[B11] Kaur R Kaur K Effects of dietary selenium on morphology of testis and cauda epididymis in rats Indian J Pharmacol 2000 44 265 272 10941613

[B12] Wu SH Oldfield JE Muth OH Whanger PD Weswig PH Effects of selenium on reproduction Proceeding Western Section, American Society of Animal Science 1969 20 85 89

[B13] Olson GE Winfrey VP Hill KE Burk RF Sequential development of flagellar defects in spermatids and epididymal spermatozoa of selenium-dificient rats Reprod 2004 127 335 342 10.1530/rep.1.00103 15016953

[B14] Kidd SA Eskenazi B Wyrobex AJ Effects of male age on semen quality and fertility: a review of the literature Fertil Steril 2001 75 237 248 10.1016/S0015-0282(00)01679-4 11172821

[B15] Mohammadi Sh Movahedin M Mowla SJ The Effects of Selenium Antioxidant Activity on Sperm Parameters and Testis Structure in Aging and Adult Male Mice J Reprod Infertil (Persian) 2008 3 26 230 238

[B16] Gan L Liu Q Xu H Zhu Y Yang XL Effects of selenium overexposure on glutathione peroxidase and thioredoxin reductase gene expressions and activities Biol Trace Element Res 2002 89 165 175 10.1385/BTER:89:2:165 12449240

[B17] Shalini S Banasal MP Role of selenium in spermatogenesis: differential expression of cjun and cfos in tubular cells of mice testis Mol cell Biochem 2006 292 27 38 10.1007/s11010-006-9168-9 17066317

